# Implementation of Artificial Intelligence–Based Diabetic Retinopathy Screening in a Tertiary Care Hospital in Quebec: Prospective Validation Study

**DOI:** 10.2196/59867

**Published:** 2024-09-03

**Authors:** Fares Antaki, Imane Hammana, Marie-Catherine Tessier, Andrée Boucher, Maud Laurence David Jetté, Catherine Beauchemin, Karim Hammamji, Ariel Yuhan Ong, Marc-André Rhéaume, Danny Gauthier, Mona Harissi-Dagher, Pearse A Keane, Alfons Pomp

**Affiliations:** 1 Institute of Ophthalmology University College London London United Kingdom; 2 Department of Ophthalmology Centre Hospitalier de l'Université de Montréal Montreal, QC Canada; 3 Department of Ophthalmology Université de Montréal Montreal, QC Canada; 4 The CHUM School of Artificial Intelligence in Healthcare Centre Hospitalier de l'Université de Montréal Montreal, QC Canada; 5 Moorfields Eye Hospital NHS Foundation Trust London United Kingdom; 6 Health Technology Assessment Unit Centre Hospitalier de l'Université de Montréal Montreal, QC Canada; 7 Division of Endocrinology Department of Medicine Centre Hospitalier de l'Université de Montréal Montreal, QC Canada; 8 Direction du soutien à la transformation Centre Hospitalier de l'Université de Montréal Montreal, QC Canada; 9 Faculty of Pharmacy University of Montreal Montreal, QC Canada; 10 Oxford Eye Hospital Oxford University Hospitals NHS Foundation Trust Oxford United Kingdom; 11 NIHR Moorfields Biomedical Research Centre London United Kingdom; 12 Department of Surgery University of Montréal Montreal, QC Canada

**Keywords:** artificial intelligence, diabetic retinopathy, screening, clinical validation, diabetic, diabetes, screening, tertiary care hospital, validation study, Quebec, Canada, vision, vision loss, ophthalmological, AI, detection, eye

## Abstract

**Background:**

Diabetic retinopathy (DR) affects about 25% of people with diabetes in Canada. Early detection of DR is essential for preventing vision loss.

**Objective:**

We evaluated the real-world performance of an artificial intelligence (AI) system that analyzes fundus images for DR screening in a Quebec tertiary care center.

**Methods:**

We prospectively recruited adult patients with diabetes at the Centre hospitalier de l’Université de Montréal (CHUM) in Montreal, Quebec, Canada. Patients underwent dual-pathway screening: first by the Computer Assisted Retinal Analysis (CARA) AI system (index test), then by standard ophthalmological examination (reference standard). We measured the AI system's sensitivity and specificity for detecting referable disease at the patient level, along with its performance for detecting any retinopathy and diabetic macular edema (DME) at the eye level, and potential cost savings.

**Results:**

This study included 115 patients. CARA demonstrated a sensitivity of 87.5% (95% CI 71.9-95.0) and specificity of 66.2% (95% CI 54.3-76.3) for detecting referable disease at the patient level. For any retinopathy detection at the eye level, CARA showed 88.2% sensitivity (95% CI 76.6-94.5) and 71.4% specificity (95% CI 63.7-78.1). For DME detection, CARA had 100% sensitivity (95% CI 64.6-100) and 81.9% specificity (95% CI 75.6-86.8). Potential yearly savings from implementing CARA at the CHUM were estimated at CAD $245,635 (US $177,643.23, as of July 26, 2024) considering 5000 patients with diabetes.

**Conclusions:**

Our study indicates that integrating a semiautomated AI system for DR screening demonstrates high sensitivity for detecting referable disease in a real-world setting. This system has the potential to improve screening efficiency and reduce costs at the CHUM, but more work is needed to validate it.

## Introduction

Diabetes mellitus is a prevalent metabolic disease affecting 5.7 million Canadians, or 14% of the population, in 2022. This number is expected to increase to 7.3 million by 2032 [1]. Diabetic retinopathy (DR) is a common complication of the disease, affecting up to 25% of patients in Canada [2]. DR is also the leading cause of vision loss in people of working age and is associated with increased mortality [3]. Detection of DR at an early stage, when it can be treated with the best prognosis, is crucial in preventing vision loss [4].

Diabetes Canada recommends annual to biennial DR screening by an ophthalmologist or an optometrist for all people with diabetes [3]. Despite this recommendation, up to a third of diabetic patients go unscreened in Ontario, for example [5]. Demographic and socioeconomic factors, such as low income and immigration, are the main risk factors for being unscreened [5]. Numerous Canadian provinces have implemented more inclusive DR screening programs through DR telescreening (DRTS) [6-10].

In Quebec, significant progress has been made in developing DRTS pathways for patients with diabetes in distant regions [11]. However, in large urban centers, only a minority of patients benefit from such access [6]. For most patients, screening is carried out in optometry clinics, resulting in out-of-pocket expenses, as these services are not covered by the provincial health insurance program [12]. Many patients are referred to consult with an ophthalmologist at a hospital. However, this care process is usually inefficient, with numerous obstacles to effective screening [6].

The recent demonstrations of artificial intelligence (AI)-based grading systems for DR have sparked interest into their integration in pre-established DRTS care pathways in Quebec [11,13]. These systems can integrate existing DRTS pathways in 2 ways: a semiautomated manner, where they replace the preliminary triage currently performed by level 1 trained graders [14]. Alternatively, they can operate in a fully autonomous way, which would not require any human oversight [15].

In this study, we share findings from incorporating a semiautomated AI model into the care strategy for diabetic patients at a major tertiary care center in Quebec. We report on the prospective clinical validation of this AI system, showcasing its real-world performance at both the patient and eye levels for detecting retinopathy and diabetic macular edema (DME). We also perform an economic analysis to estimate the potential cost savings achievable with the implementation of the AI system.

## Methods

### Study Design

The Centre hospitalier de l’Université de Montréal (CHUM) is a tertiary and quaternary care teaching hospital and one of the two major health care networks in Montreal, Quebec, Canada. It serves 500,000 patients annually. At the CHUM, patients with diabetes are managed by the endocrinology service in specialized clinics, with DR screening conducted either externally or within the ophthalmology department, depending on patient and physician choice.

**Figure 1:**
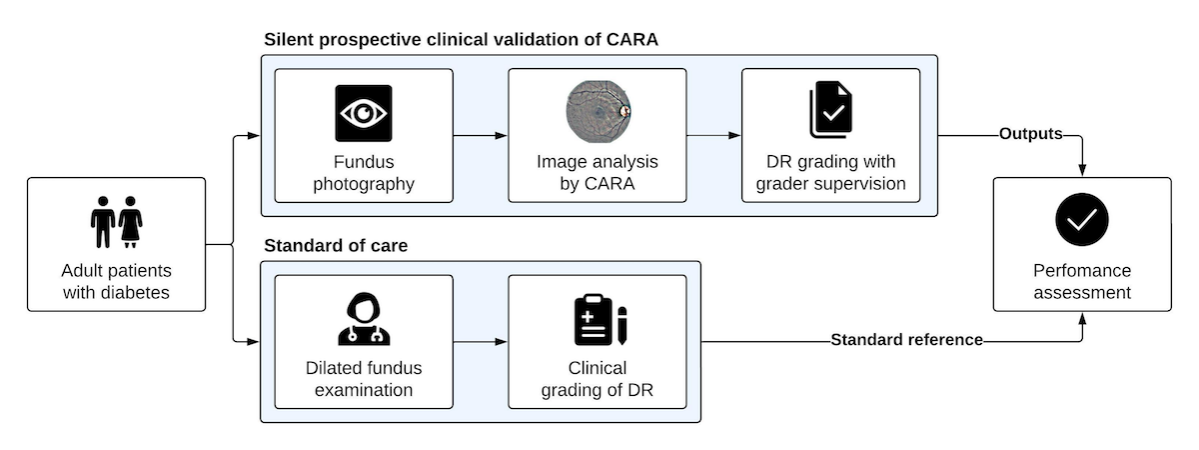
Overview of the silent prospective clinical validation study. Adult patients with diabetes were recruited from CHUM's endocrinology service. After consenting, fundus photography was obtained on the same day. The fundus images were then transferred through the cloud to the prospective validation pipeline. The images were automatically analyzed by the CARA algorithm. Before generating the report, a trained grader employed by the service provider would grade the CARA-processed images. Within a month, with the outputs of CARA masked, the patients were referred to receive standard of care screening with an ophthalmologist at the CHUM. To determine the discriminative performance of CARA, the outputs from the silent prospective validation were compared to the ophthalmologist exam (standard reference). CARA: Computer Assisted Retinal Analysis; CHUM: Centre hospitalier de l’Université de Montréal; DR: diabetic retinopathy.

To improve the patient experience and increase screening uptake, we implemented an AI-based DRTS pathway within the CHUM. To evaluate our approach, we designed a silent prospective clinical validation trial. All recruited patients were screened through 2 parallel pathways: screening by an AI-based system called Computer Assisted Retinal Analysis (CARA), followed by standard of care screening by a CHUM ophthalmologist who was masked to the output of CARA. The study pathway is described in Figure 1.

### Study Population

Patients who attended the diabetes clinic of the endocrinology service between September 2018 and January 2019 were recruited. All patients were new patients to the department of ophthalmology. Previous DR diagnosis, past retinal disease, or intraocular surgery was not exclusionary.

### CARA: A Semiautomated AI Model for DR Detection

The CARA system, a semiautomated AI developed by Diagnos Inc. in Montreal, is a clinically inspired machine learning (ML) algorithm designed to detect retinopathy and maculopathy. As a traditional ML model, CARA required the handcrafting of feature extractors that transform pixel values of a retinal image into feature vectors for an ML classifier [16,17]. This is different from deep learning approaches which became prominent after the development of CARA [17,18]. CARA identifies specific lesions in retinal images, including dark lesions like microaneurysms and hemorrhages, as well as bright lesions such as hard exudates and cotton wool spots [19,20].

The algorithm uses a multistep process involving image enhancement, laterality determination, vascular network, optic nerve and fovea detection, and lesion identification. A final output is reached by a weighted combination of image quality, the highest probability of bright and dark lesions, and image conformity. This output is reviewed by a Diagnos Inc senior grader before being sent to the CHUM, marking the process as semiautomated. The senior grader, an ophthalmologist with 12 years of grading experience, was employed by Diagnos Inc. The CHUM was not informed of any formal auditing or quality assessment methods used by Diagnos Inc.

CARA received approval from the US Food and Drug Administration as a class II medical device in 2011 through the 510(k) pathway. It was intended as a software platform to visualize, store, and enhance color fundus images through computerized networks, but was not certified for diagnosing DR at that time. Prior to this current study, CARA had shown a 93% sensitivity and 71% specificity in retinopathy detection across an international data set of 509 eyes (personal communication with Diagnos Inc). Our study served as a first prospective clinical validation study for the CARA service.

### Fundus Photography

On recruitment day, in the endocrinology clinic, 45-degree, nonstereoscopic color fovea-centered fundus photographs were obtained by a technician, without pupillary mydriasis, using the Centervue DRS camera (Hillrom, Chicago, United States). A single image was obtained per eye as CARA was designed to make classifications from single-field images. At the time of the study's design, there were no Canadian recommendations in that regard, but this approach seemed reasonable as single-field images were being used in the Scottish DR screening services, for example [21]. The acquired images were uploaded to the CARA platform for them to be processed by the ML algorithm. The generation of AI outputs was carried out asynchronously and the patient, the referring endocrinologist, and all eye care providers were masked to the output of the AI system.

### Ophthalmologist Examination (Standard Reference)

Within a month of recruitment, patients were referred to undergo screening by an ophthalmologist using slit lamp examination, typically with a 78 or 90D lens. Fundus photography and optical coherence tomography were not routinely carried out. Each patient's grading was performed by a single attending ophthalmologist, with the study including a total of 28 ophthalmologists with diverse subspecialties and levels of experience (Multimedia Appendix 1). No specific grading training was administered; however, all participating ophthalmologists were board-certified by the Royal College of Physicians and Surgeons of Canada. They were expected to have the competence to diagnose and grade DR, as it is considered an objective (#3.1.2.4.2.2) of ophthalmology training by the Royal College [22]. Using standardized case report forms, the ophthalmologists graded each eye using the Scottish Diabetic Retinopathy Grading Scheme [23,24]. Standardized imaging examples were available as needed. This represented the standard reference to which the AI model was compared to.

### Disease Definition

In a DR screening program, individuals with no or mild DR undergo annual monitoring, while those with more than mild (mtmDR), and those with DME are referred for evaluation [25]. Accordingly, numerous pivotal trials of AI-based DR screening models have focused on their ability to detect mtmDR [13,15]. The intended use of CARA, however, was as a tool to detect any retinopathy (including mild disease) and DME. This included, according to the Scottish Diabetic Retinopathy Grading Scheme: R1, R2, R3, and R4 for retinopathy grading, and M1 and M2 for DME grading [23]. This is equivalent to level 1 triaging responsibility as defined by the CR2N Tele-Retina Steering Committee [14].

For each patient, we determined if they were referable by mapping the retinopathy and DME labels to “retinopathy or DME present” (referable) or “retinopathy and DME absent” (not referable), taking the worst of the 2 eyes to correspond to the outputs of the AI system at the patient level. Patients without referral outputs for both eyes were considered “inconclusive.”

### Economic Analysis

We conducted an economic evaluation to estimate the costs of adopting the CARA system for screening 5000 annual patients with diabetes at the CHUM, analyzed from a health care system perspective. The direct costs of screening, and costs for inconclusive outputs and false positive referrals were considered in the analysis. All values are presented in Canadian dollars.

### Outcomes and Statistical Analysis

The primary outcomes were the sensitivity and specificity of the AI system to detect referable disease at the patient level. The secondary outcomes were first, the sensitivity and specificity of the AI system for the detection of retinopathy and DME at the eye level, and second, the cost savings in Canadian dollars should the system be implemented.

Sensitivity, specificity, positive predictive value, and negative predictive value for each of retinopathy and DME detection were computed from the confusion matrices. We used the Wilson score interval method to calculate 95% CI. The study follows the Standards for Reporting of Diagnostic Accuracy (STARD) checklist [26].

### Ethical Considerations

Patients were enrolled only if they understood the study and provided informed consent. This research complied with the Declaration of Helsinki. Departmental approval was obtained, and an ethical waiver was granted by the CHUM given the silent, noninterventional nature of the study. All data was deidentified.

## Results

### Patient Flow

**Figure 2:**
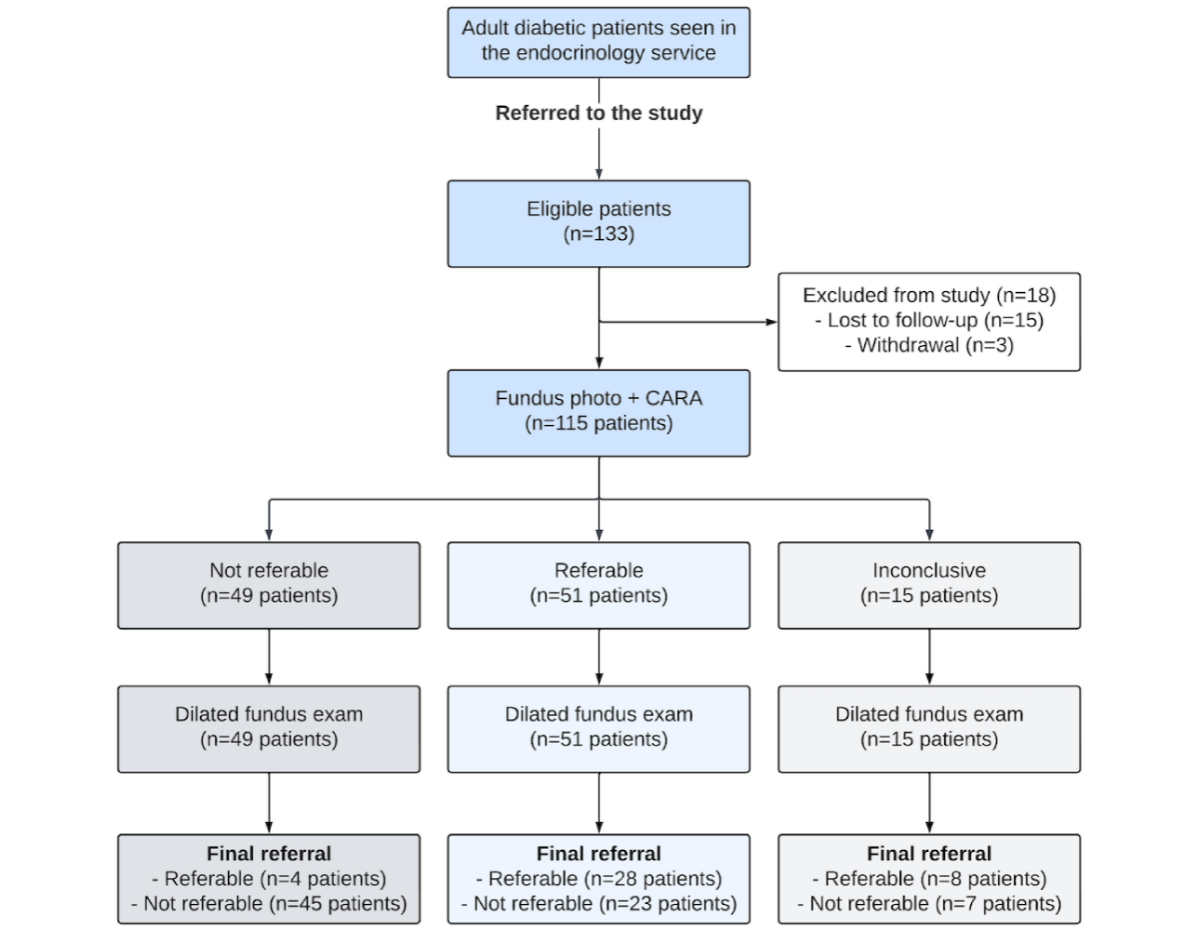
STARD (Standards for Reporting of Diagnostic Accuracy) flowchart at the patient level. Referable disease was defined as “retinopathy or DME present” and not referable was defined as “retinopathy and DME absent”, taking the worst of the two eyes to correspond to the outputs of the AI system at the patient level. CARA: Computer Assisted Retinal Analysis; DME: diabetic macular edema.

**Figure 3:**
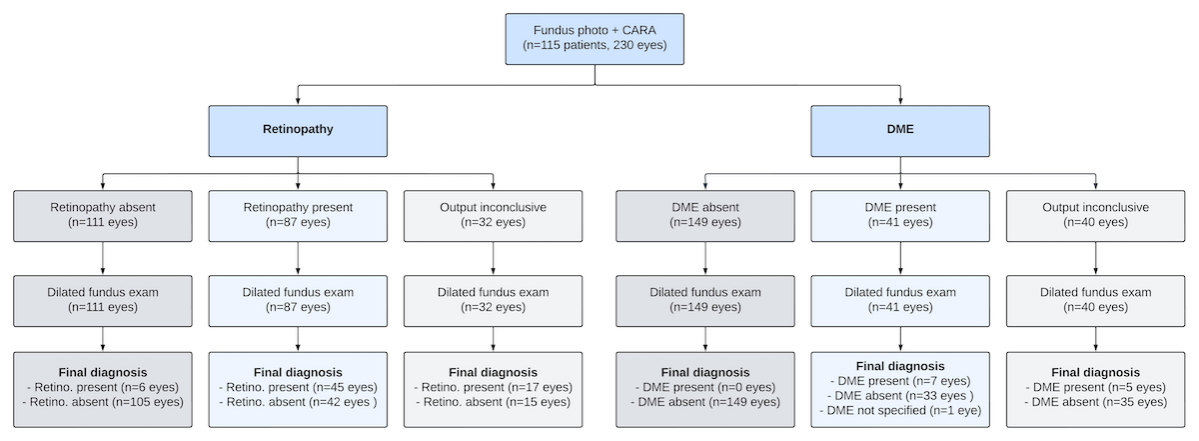
STARD (Standards for Reporting of Diagnostic Accuracy) flowchart at the eye level. Using the Scottish Diabetic Retinopathy Grading Scheme, retinopathy was defined as the presence of any retinopathy (corresponding to R1, R2, R3, and R4). DME was defined as the presence of M1 or M2. CARA: Computer Assisted Retinal Analysis; DME: diabetic macular edema.

Between September 2018 and January 2019, the endocrinology service referred 133 patients to the study, of which 115 (230 eyes) underwent fundus imaging and received CARA outputs. Multimedia Appendix 2 presents demographics of the 18 excluded patients, showing no significant differences from those included. Figures 2 and 3 illustrate recruitment at the patient and eye levels for retinopathy and DME, respectively.

### Study Cohort

Patient demographics and characteristics are summarized in Table 1. The majority of patients were male (57.4%) with a mean age of 55.4 (SD 15.6) years. Most patients were White (60.9%). While the type of diabetes was the most often unspecified (43.5%), type 2 diabetes (36.5%) was more common than type 1 (20.0%).

**Table 1:** Patient demographics for the patients included in the study.

Demographic		Value (N=115)
**Sex, n (%)**		
	Male	66 (57.4)
	Female	49 (42.6)
**Age (years)**		
	Mean (SD)	55.4 (15.6)
	Range	20-90
**Diabetes type, n (%)**		
	Type 1	23 (20)
	Type 2	42 (36.5)
	Unspecified^a^	50 (43.5)
**Ethnicity, n (%)**		
	White	70 (60.9)
	Middle-Eastern	17 (14.8)
	Hispanic	13 (11.3)
	Black	11 (9.6)
	Unknown	4 (3.5)

^a^Diabetes subtype not specified on the study referral form.

### Performance for Detecting Referable Patients

**Figure 4:**
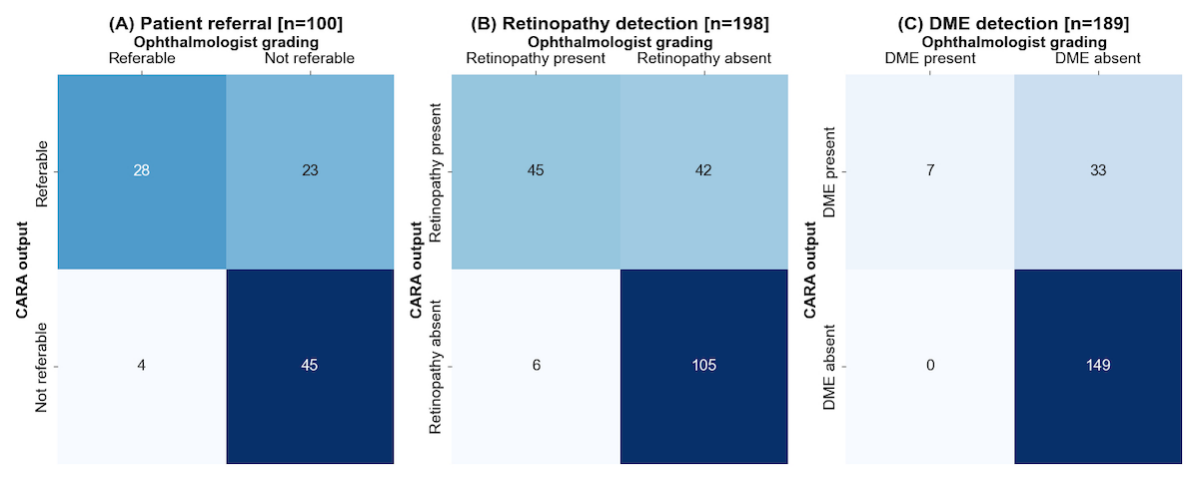
Confusion matrices showing the discriminative performance of CARA (Computer Assisted Retinal Analysis) at the patient level (referral) and eye level (retinopathy and DME [diabetic macular edema] detection) for patients with analyzable outputs. At the patient level (n=115), 100 had analyzable outputs, and 15 were inconclusive. At the eye level, from 230 eyes, 32 and 40 eyes had inconclusive AI outputs for retinopathy and DME, respectively, and 1 eye lacked an ophthalmologist's grading (DME). This resulted in 198 and 189 eyes with analyzable outcomes for retinopathy and DME, respectively.

The primary outcome was model performance at the patient level. For the 100 patients with analyzable data, the confusion matrix demonstrating the performance of CARA for referable patient detection is shown in Figure 4A. CARA had a sensitivity of 87.5% (95% CI 71.9-95.0) and a specificity of 66.2% (95% CI 54.3-76.3). There were 4 false negatives with mild background retinopathy (R1) not requiring treatment. No cases of vision-threatening retinopathy were missed.

Inconclusive outputs occurred in 13% (15/115) patients. Reasons varied, including imageability issues like small pupil (n=5), uncooperative patient (n=1), and media opacity (n=2), along with ungradable model outputs due to processing errors or unmet decision thresholds (n=7). Multimedia Appendix 3 describes the demographics of this patient cohort, noting older age in patients with inconclusive outputs (69.5 vs 53.3, *P*<.001). In this group, 8 patients (53.3%) had referable disease, including 3 cases of DME but no cases of severe nonproliferative or proliferative retinopathy.

### Performance for Detecting Disease at the Eye Level

The confusion matrix demonstrating the performance of CARA for retinopathy detection is shown in Figure 4B. CARA had a sensitivity of 88.2% (95% CI 76.6-94.5) and a specificity of 71.4% (95% CI 63.7-78.1). Among the 32 eyes with an inconclusive CARA output for retinopathy, 17 eyes had retinopathy (53.1%) and 15 did not (46.9%).

The confusion matrix demonstrating the performance of CARA for DME detection is shown in Figure 4C. CARA had a sensitivity of 100% (95% CI 64.6-100) and a specificity of 81.9% (95% CI 75.6-86.8). The remaining performance metrics are summarized in Table 2. Among the 40 eyes with an inconclusive CARA output for DME, 5 eyes had DME (12.5%) and 35 did not (87.5%).

**Table 2:** Summary of the discriminative performance of CARA^a^.

Metric	Patient level	Eye level	
	Referable disease	Retinopathy	DME^b^
Sensitivity (95% CI)	87.5 (71.9-95.0)	88.2 (76.6-94.5)	100.0 (64.6-100)
Specificity (95% CI)	66.2 (54.3-76.3)	71.4 (63.7-78.1)	81.9 (75.6-86.8)
PPV^c^ (95% CI)	54.9 (41.4-67.7)	51.7 (41.4-61.9)	17.5 (8.8-32.0)
NPV^d^ (95% CI)	91.8 (80.8-96.8)	94.6 (88.7-97.5)	100.0 (97.5-100)

^a^CARA: Computer Assisted Retinal Analysis

^b^DME: Diabetic macular edema.

^c^PPV: Positive predictive value (precision).

^d^NPV: Negative predictive value.

### Economic Analysis

A detailed description of the assumptions and calculations performed for the economic analysis is described in Multimedia Appendix 4**.** Implementing the AI system for screening could result in a yearly savings of CAD $245,635 (US $177,643.23, as of July 26, 2024) or CAD $49 (US $35.44) per patient, as shown in Table 3.

These estimates are based on 5000 patients followed annually at the diabetes clinic of the CHUM. The calculations are based on specific costs per patient, the prevalence of diabetic retinopathy among analyzable cases, the percentage of inconclusive outputs by the AI system, and the AI system's specificity. The final row highlights the total cost savings achievable when employing the AI system for screening, demonstrating a significant economic advantage over the current standard of care.

**Table 3:** Cost comparison between standard of care and AI^a^ system screening for DR^b^ screening.

Cost component (CAD $^c^)	Standard of care	AI system
Direct screening cost	590,500	150,000
Cost for inconclusive outputs	N/A^d^	76,765
Cost for false positive referrals	N/A	118,100
Total cost	590,500	344,865
Cost savings	N/A	245,635
Average cost per patient	118	69
Average cost savings per patient	N/A	49

^a^AI: Artificial intelligence

^b^DR: Diabetic retinopathy

^c^As of July 26, 2024, CAD $1=US $0.7232)

^d^N/A: not applicable.

## Discussion

### Principal Findings

AI-based telescreening is a promising approach that has the potential to improve community-based screening of DR [27,28]. When effective, AI-based DRTS systems can help reduce unnecessary examinations of patients without DR, allowing resource allocation to those who need more active management [28]. In this work, we describe the outcomes of real-world implementation of an AI-based DRTS for the screening of DR in a tertiary care hospital in Montreal.

We carried out a silent prospective clinical validation study to assess the performance of the CARA system prior to its implementation in our local population [29]. Our cohort featured an equitable sex distribution and a strong representation of several visible minority groups in Montreal (Black, Middle-Eastern, and Hispanic), which makes our findings generalizable to the local population [30]. The retention rate in this study was notably high, with 86.5% completing the study. While this hints at positive patient acceptance, further studies will be needed for a conclusive understanding of patient acceptability of this technology. The literature suggests a general preference among patients for automated DR screening [31,32], especially when clinicians are involved in supervising the AI system's decisions [33].

CARA identified patients for referral with 87.5% sensitivity and 66.2% specificity. It detected retinopathy with 88.2% sensitivity and 71.4% specificity, and DME with 100% sensitivity and 81.9% specificity. These results meet the 85% sensitivity benchmark obtained by several similar AI systems designed for DR screening [34]. In the real-world setting, a recent meta-analysis showed a pooled sensitivity of 91% (95% CI 87-94) and specificity of 92% (95% CI 88-94) for detecting any retinopathy at the eye level [35]. For vision-threatening DR, which includes DME, sensitivity was 99% (95% CI 95-100) and specificity 92% (95% CI 74-98) [35]. CARA's performance in detecting retinopathy and DME is on par with other systems in sensitivity but not in specificity, suggesting it effectively identifies disease needing referral but may result in higher false positive rates than desired. The performance gap between CARA and the current state of the art may be partly due to the inherent limitations of traditional feature-based models like CARA [36,37]. In contrast, novel neural network–based models have seen significant improvements in recent years, driven by enhanced architectures and the availability of extensive training data sets [38].

**Figure 5:**
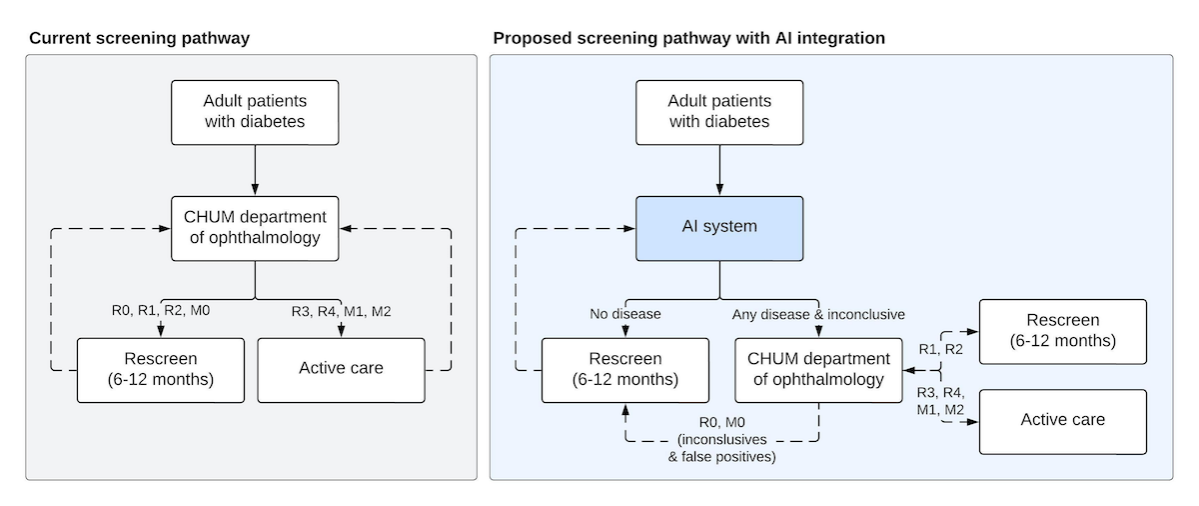
Proposed clinical pathway for the artificial intelligence (AI) system implementation. In the current screening pathway, all patients are evaluated by the department of ophthalmology. With AI system integration as a level 1 triage agent, disease-free patients would be filtered out, reducing unnecessary referrals. Only those with referable disease or an inconclusive AI output would be directed to ophthalmology. False positives would re-enter the AI screening pathway, while patients with any disease receive follow-up care in ophthalmology. CHUM: Centre hospitalier de l’Université de Montréal.

Despite the limited specificity, CARA could significantly reduce the workload of the ophthalmology department at the CHUM by filtering out patients without DR (Figure 5). While false positives can create patient stress and costs [39], since ophthalmologists evaluate these cases, they are unlikely to lead to unnecessary treatments. A similar approach has been successful in the Scottish DRTS program, where all images undergo a first pass through an “autograder” algorithm [40]. With similar specificity to CARA, the Scottish system’s automated grading has been shown to be safe [41], and has reduced the burden of manual grading by up to one-third [42]. The current CARA service, in its semiautomated setup, however, still requires grader oversight. Despite that, we expect it could also reduce the burden of manual grading.

Implementing CARA in our screening pathway could yield an estimated first-year saving of approximately CAD $245,635 (US $177,643.23) for 5000 patients (CAD $49 [US $35.44] per patient), considering direct screening costs and those related to false positive and inconclusive outputs. Of note, the potential costs associated with false negatives (missed cases) were not considered in the analysis due to the lack of such data in our context. The impact of the CARA system on quality-adjusted life years also was not assessed. In the future, we will focus on modeling the screening pathway with decision trees to calculate the incremental cost-effectiveness of our approach, also accounting for false negative cases.

Our study has several limitations and design flaws that have become identifiable in hindsight. Firstly, the provision of the CARA AI system as a service provided to the CHUM introduced several constraints to our study. We lacked control over the model both before and during its implementation. The final outputs were sent to us post grader assessment, without insights into model failures and ungradable images. The semiautomated nature of the pipeline meant that the performance metrics could have been influenced by grader performance, which we are now unable to measure retrospectively. Secondly, we employed dilated fundus examinations as the reference standard instead of using image-assisted evaluations with fundus photographs and optical coherence tomographic scans. This screening method, common in Quebec, might not capture the full human diagnostic accuracy achievable through a multimodal approach. Due to these limitations, our findings should not be used as a basis to support the approval of CARA as a software medical device. Instead, this study should be viewed as a preliminary trial that helped develop pathways for future, more robust studies involving AI in the DR screening process at CHUM.

Building on those learnings, we have designed a new clinical trial to evaluate the real-world performance of a fully automated deep learning system called NeoRetina (Diagnos Inc) [43]. This new model uses neural networks, which represent the current state of the art in AI. To have a more robust standard reference, in addition to the routine ophthalmological evaluation of DR and DME, masked grading of the same retinal photographs used by NeoRetina will be performed. These images will be assessed for quality and then graded by at least 2 fellowship-trained retina specialists, with a predefined arbitration process. The new trial will leverage the screening pathways developed in this study and will aim to recruit 630 patients by December 2026.

### Conclusions

In conclusion, we report findings from the first real-word implementation study of an AI-based DRTS system in the province of Quebec and possibly in Canada [35]. Large-scale implementation of CARA at CHUM would be expected to result in cost savings and reduced waiting times. However, we are currently investigating more advanced models that we aim to validate more robustly. Once deployed, any model will require routine audits to ensure model performance is maintained, especially with changes in population demographics and disease patterns over time [44,45]. Similarly, ensuring strict information governance policies will be crucial for protecting patient data and responsibly leveraging the benefits of AI systems.

## Appendix

Multimedia Appendix 1 Subspecialties, years of experience of the grading ophthalmologists, and numbers of images graded.

Multimedia Appendix 2 Patient demographics of the 18 patients who were excluded from the study. After recruitment, 15 patients were lost to follow-up and 3 patients withdrew. There were no statistically significant differences in the patient demographics. *The comparison between the study cohort (n=115) and excluded group (n=18) was performed using Mann-Whitney U test for all continuous variables. For categorical variables, we used the Chi-squared test. **Diabetes subtype not specified on the study referral form.

Multimedia Appendix 3 Patient demographics of the 15 patients with inconclusive Computer Assisted Retinal Analysis (CARA) outputs. The cohort of patients with inconclusive CARA outputs was significantly older than the one with analysable outputs (*P*< 0.001). *The comparison between the cohort with analysable outputs (n=100) and the group with inconclusive outputs (n=15) was performed using Mann-Whitney U test for all continuous variables. For categorical variables, we used the chi-squared test. **Diabetes subtype not specified on the study referral form.

Multimedia Appendix 4 Cost parameters, assumptions and calculations for the economic analysis. AI: artificial intelligence; FPR: false positive rate: pt: patient.

## Abbreviations

AI: artificial intelligence
CARA: Computer Assisted Retinal Analysis
CHUM: Centre hospitalier de l’Université de Montréal
DME: diabetic macular edema
DR: diabetic retinopathy
DRTS: DR telescreening
ML: machine learning
STARD: Standards for Reporting of Diagnostic Accuracy
