# Supplementary material for: Implementation of Artificial Intelligence–Based Diabetic Retinopathy Screening in a Tertiary Care Hospital in Quebec: Prospective Validation Study
Source: JMIR Diabetes. 2024 Sep 3;9:e59867. doi: 10.2196/59867 (PMC11408885; doi:10.2196/59867)
Supplement: Multimedia Appendix 1 [file diabetes_v9i1e59867_app1.pdf]

**Supplementary table 1. Subspecialties, years of experience of the grading ophthalmologists, and numbers of images graded.**

| **Subspecialty** | **Number of ophthalmologists (n=28)** | **Number of patients graded per specialty (n=115)** |
|---|---|---|
| Low vision | 1 | 4 (3.5%) |
| Cornea | 5 | 19 (16.5%) |
| Glaucoma | 4 | 13 (11.3%) |
| Neuro-ophthalmology | 4 | 17 (14.8%) |
| Oculoplastics | 1 | 3 (2.6%) |
| Ocular oncology | 1 | 1 (0.9%) |
| General ophthalmology | 2 | 12 (10.4%) |
| Retina | 7 | 36 (31.3%) |
| Strabismus | 1 | 3 (2.6%) |
| Uveitis | 2 | 7 (6.1%) |
| **Years of experience** | | |
| Mean (SD) | 16.9 (13.1) | |
| Range | 3 - 51 | |
| **Number of patients graded per ophthalmologist** | | |
| Mean (SD) | 4.1 (1.7) | |
| Median (IQR) | 4 (2) | |
| Range | 1 - 9 | |
