# Supplementary material for: Implementation of Artificial Intelligence–Based Diabetic Retinopathy Screening in a Tertiary Care Hospital in Quebec: Prospective Validation Study
Source: JMIR Diabetes. 2024 Sep 3;9:e59867. doi: 10.2196/59867 (PMC11408885; doi:10.2196/59867)
Supplement: Multimedia Appendix 2 [file diabetes_v9i1e59867_app2.pdf]

Supplementary table 2. Patient demographics of the 18 patients who were excluded from the study. After recruitment, 15 patients were lost to follow-up and 3 patients withdrew. There were no statistically significant differences in the patient demographics.

*The comparison between the study cohort (n=115) and excluded group (n=18) was performed using Mann-Whitney U test for all continuous variables. For categorical variables, we used the Chi-squared test. **Diabetes subtype not specified on the study referral form.

**Demographic**		**Excluded (n=18)**	**Study cohort (n = 115)**	**P-value***
**Sex**	Male	8 (44.4%)	66 (57.4%)	0.304
	Female	10 (55.6%)	49 (42.6%)	
**Age**	Mean (SD)	58.6 (15.2)	55.4 (15.6)	0.308
	Range	29 - 76	20 - 90	
**Diabetes type**	Type 1	3 (16.7%)	23 (20.0%)	0.629
	Type 2	5 (27.8%)	42 (36.5%)	
	Unspecified**	10 (55.6%)	50 (43.5%)	
**Ethnicity**	White	14 (77.8%)	70 (60.9%)	0.451
	Middle-Eastern	3 (16.7%)	17 (14.8%)	
	Hispanic	0 (0%)	13 (11.3%)	
	Black	1 (5.6%)	11 (9.6%)	
	Unknown	0 (0%)	4 (3.5%)	
