# Supplementary material for: Implementation of Artificial Intelligence–Based Diabetic Retinopathy Screening in a Tertiary Care Hospital in Quebec: Prospective Validation Study
Source: JMIR Diabetes. 2024 Sep 3;9:e59867. doi: 10.2196/59867 (PMC11408885; doi:10.2196/59867)
Supplement: Multimedia Appendix 3 [file diabetes_v9i1e59867_app3.pdf]

Supplementary table 3. Patient demographics of the 15 patients with inconclusive CARA outputs. The cohort of patients with inconclusive CARA outputs was significantly older than the one with analysable outputs ($p < 0.001$).

*The comparison between the cohort with analysable outputs (n=100) and the group with inconclusive outputs (n=15) was performed using Mann-Whitney U test for all continuous variables. For categorical variables, we used the Chi-squared test. **Diabetes subtype not specified on the study referral form.

**Demographic**		**Inconclusive (n=15)**	**Analysable (n = 100)**	**P-value***
**Sex**	Male	9 (60.0%)	57 (57.0%)	0.827
	Female	6 (40.0%)	43 (43.0%)	
**Age**	Mean (SD)	69.5 (15.3)	53.3 (14.5)	**<0.001**
	Range	28 - 90	20 - 83	
**Diabetes type**	Type 1	0 (0%)	23 (23.0%)	0.101
	Type 2	6 (40.0%)	36 (36.0%)	
	Unspecified**	9 (60.0%)	41 (41.0%)	
**Ethnicity**	White	7 (46.7%)	63 (63.0%)	0.303
	Middle-Eastern	2 (13.3%)	15 (15.0%)	
	Hispanic	4 (26.7%)	9 (9.0%)	
	Black	1 (6.7%)	10 (10.0%)	
	Unknown	1 (6.7%)	3 (3.0%)	
