# Supplementary material for: Implementation of Artificial Intelligence–Based Diabetic Retinopathy Screening in a Tertiary Care Hospital in Quebec: Prospective Validation Study
Source: JMIR Diabetes. 2024 Sep 3;9:e59867. doi: 10.2196/59867 (PMC11408885; doi:10.2196/59867)
Supplement: Multimedia Appendix 4 [file diabetes_v9i1e59867_app4.pdf]

Supplementary table 4. Cost parameters, assumptions and calculations for the economic analysis.

Abbreviations: AI, artificial intelligence; FPR, false positive rate; pt, patient

**Cost parameter**	**Assumption**	**Total amount for 5,000 patients**
**Direct screening cost - Standard of care**	Clinic running costs: CAD 44.70/ pt; physician remuneration: CAD 73.40/ pt; total = CAD 118.10/ pt	CAD 118.10/ pt x 5,000 pts = CAD 590,500
**Direct screening cost - AI system**	Negotiated rate: CAD 30.00/ pt	CAD 30.00/ pt x 5,000 pts = CAD 150,000
**Cost for inconclusive outputs by the AI system**	In the study, 15/115 patients had inconclusive results (13%).<br><br>Projection: 13% of 5,000 future patients may have similar results = 650 patients These patients would require a standard ophthalmologist examination.	CAD 118.10/ pt x 650 pts = CAD 76,765
**Cost for false positive referrals**	**False positive rate**<br>FPR = *1 − specificity*<br>With a specificity of 66.2%, the FPR is 1 − 0.662 = 33.8%.<br>This rate is pertinent to individuals who do not have referable disease.<br><br>**Number of pts with non referable disease**<br>Nb of pts with non referable disease = *1 − prevalence of referable disease.*<br>With a prevalence of 32% among analysable cases, this results in 1 − 0.32 = 68%.<br>This number applies to patients with analysable outcomes, projected to be 4,350 (subtracting 650 inconclusive cases from 5,000).<br>Applying 68% to 4,350 patients, we anticipate 0.68 × 4,350 = 2,958 to be disease-free.<br><br>**Number of patients with a false positive result**<br>By applying the FPR to this population, we estimate that<br>33.8% × 2,958 = 999.8 (≈1,000) patients without the disease would receive a false positive result.	CAD 118.10/ pt x 1,000 pts = CAD 118,100
